# Abatement of potent P2Y12 antagonist-based dual antiplatelet therapy after coronary intervention: A network meta-analysis of randomized controlled trials

**DOI:** 10.3389/fcvm.2022.1008914

**Published:** 2023-01-12

**Authors:** Oumaima El Alaoui El Abdallaoui, Dániel Tornyos, Réka Lukács, András Komócsi

**Affiliations:** ^1^Doctoral School of Health Sciences, Faculty of Health Sciences, University of Pécs, Pécs, Hungary; ^2^Department of Interventional Cardiology, Heart Institute, Medical School, University of Pécs, Pécs, Hungary

**Keywords:** ticagrelor, prasugrel, network meta-analysis, coronary intervention, P2Y12 de-escalation therapy

## Abstract

**Introduction:**

Dual antiplatelet therapy (DAPT) including prasugrel or ticagrelor is recommended in patients with acute coronary syndromes (ACS) treated with coronary intervention (PCI). Acknowledging the importance of bleeding, multiple trials tested abatement schemes including uniform or guided de-escalation from the potent P2Y12 inhibitor (P2Y12-De) or P2Y12 inhibitor monotherapy (P2Y12-Mo) with heterogeneous results. We aimed to perform a systematic review and network meta-analysis of the impact of DAPT abatement strategies in patients with PCI.

**Methods:**

Electronic databases were searched for relevant randomized clinical studies evaluating clinical outcomes of patients after PCI. The rate of adverse events was evaluated using a frequentist network metanalysis. The random-effects model was used to combine risk estimates across trials and risk ratio (RR) with 95% confidence intervals (95% CIs) served as summary statistics. The primary endpoints of interest were the rate of major cardiac adverse events (MACE, defined as the composite of cardiovascular mortality, myocardial infarction and stroke) and bleeding.

**Results:**

Ten studies were identified randomizing 42511 patients. 6359 switched to the P2Y12-De and 13062 switched to the P2Y12-Mo. The risk of MACE, reflected a 24% reduction in the P2Y12-De and a 14% in the P2Y12-Mo in comparison with the DAPT strategy using potent P2Y12 inhibitors (RR: 0.76 [0.62, 0.94], and RR: 0.86 [0.75, 0.99], *p* < 0.05 both). A 35% risk reduction of major bleeding was seen with monotherapy (RR: 0.65 [0.46, 0.91],) contrasting the de-escalation trials where this effect was not significant (RR: 0.84 [0.57, 1.22]). All bleeding and minor bleeding events were reduced with both strategies. Indirect P2Y12-Mo versus P2Y12-De comparisons exhibited them as similar alternatives without significant differences.

**Conclusion:**

Our analysis suggests that both P2Y12-De and P2Y12-Mo reduce ischemic events and bleeding among PCI-treated ACS patients. Ischemic benefit was more expressed with P2Y12-De, however, reduction of major bleeding was only significant with P2Y12-Mo strategy.

**Systematic review registration:**

https://www.crd.york.ac.uk/prospero/display_record.php?ID=CRD42021258502, identifier CRD42021258502.

## Introduction

P2Y12 inhibitors are routinely administrated, in addition to aspirin, to reduce thrombotic complications of patients with acute coronary syndrome (ACS) undergoing percutaneous coronary intervention (PCI). Recent guidelines support the preferential use of the potent inhibitors, prasugrel or ticagrelor, as they showed a better reduction of ischemic events in their respective pivotal trials, as compared to the less effective clopidogrel (1, 2). However, these benefits come with disadvantages such as a higher risk of bleeding or side effects that may undermine patient compliance. Therefore, as observational data reflect, P2Y12 inhibitors are frequently switched during treatment in patients with ACS (3). Early after an ACS event, the higher thrombotic risk may outweigh the bleeding risk, whereas, during the chronic phase, the decrease in thrombotic risk is more pronounced than that in the bleeding risk. Abatement strategies include uniform or guided de-escalation to a less potent P2Y12 inhibitor or early cessation of aspirin and the use of potent P2Y12 inhibitor monotherapy. In addition to the pharmacological contribution to bleeding avoidance strategies, these schemes may offer potential economic benefits and, thus, are commonly practiced (4).

Nevertheless, de-escalation of antiplatelet therapy from a potent P2Y12 inhibitor may account for the large response variability of clopidogrel and the consequential issue of high on-treatment platelet reactivity (HPR), which appears in a substantial proportion of patients with ACS. Part of this response variation is explainable by genetic variations, such as the CYP2C19^*^2 and CYP2C19^*^3 loss-of-function alleles. In patients without these alleles, clopidogrel has shown a similar efficacy to those of ticagrelor and prasugrel (5). Platelet function testing (PFT) or genetic testing may, thus, make de-escalation safer by identifying patients with characteristics exposing them to an increased risk of thrombotic events and selectively maintaining potent P2Y12 inhibition for these cases (6).

Recently, multiple randomized trials were performed to test different abatement schemes. However, these were typically underpowered in order to accurately assess the efficacy and safety. Moreover, both strategies represent a potentially mutually exclusive alternative. They were tested against conventional long-term potent P2Y12 inhibitor-based DAPT treatment; however, data is lacking regarding their comparison. We aimed to evaluate the clinical outcomes of P2Y12 inhibitor de-escalation and P2Y12 inhibitor monotherapy compared with continuation of DAPT in patients treated with PCI, as well as to perform a systematic review and network meta-analysis in order to achieve greater statistical power and more precise effect estimates of the impact of DAPT abatement strategies in patients undergoing coronary intervention.

## Methods

### Search strategy

This systematic review was performed as per the standards outlined in the PRISMA Extension Statement for Reporting of Systematic Reviews Incorporating Network Meta-analyses of Healthcare Interventions (7) and was registered in the International Prospective Register of Systematic Reviews (PROSPERO; CRD42021258502).

The data that support the findings of this analysis are available from the corresponding author upon reasonable request.

### Study selection

**Figure 1:**
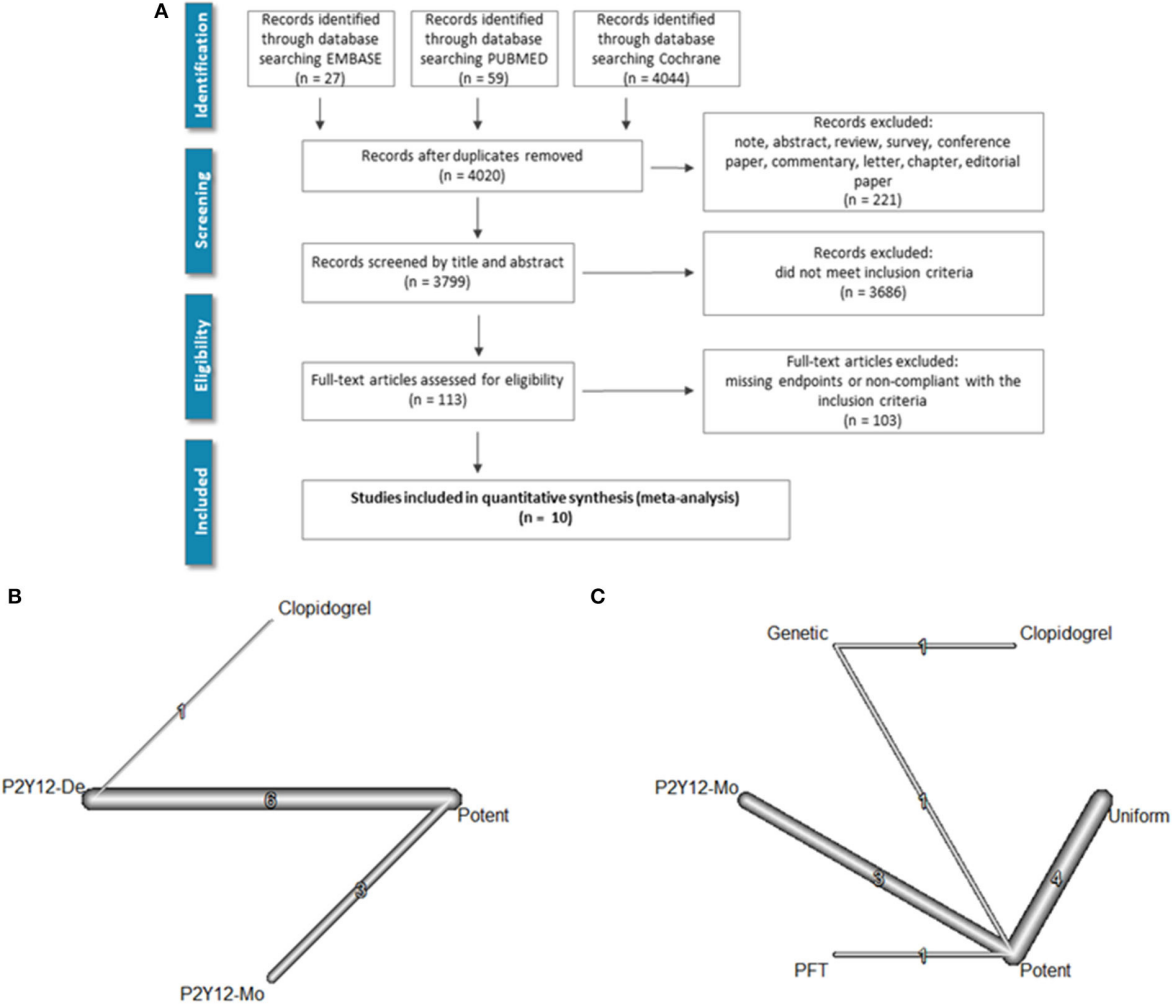
Literature search and evidence network. PRISMA flowchart of the literature search is shown in **(A)**. **(B)** Depicts the evidence network as a network of P2Y12 de-escalation (P2Y12-De), potent P2Y12 monotherapy (P2Y12-Mo), and dual antiplatelet therapy controls [i.e., DAPT with potent P2Y12 inhibitors (potent) or with clopidogrel]. In **(C)**, the network with subgrouping of de-escalation strategies is presented. De-escalation studies used three strategies either switching back to less potent P2Y12 inhibitor in every patient (uniform) or only selective de-escalation based on platelet function (PFT) or genetic characteristics (genetic).

A keyword-based search for relevant articles was performed in PubMed (MEDLINE), EMBASE, and the Cochrane Library from January 2007 to October 2021. No language restriction was used. The query included the following medical subject heading (MeSH) terms which were linked with Boolean operators: “coronary artery disease” [MeSH] OR “acute coronary syndrome” [MeSH] OR “cardiovascular disease” [MeSH] AND “de-escalation” [MeSH] AND “ticagrelor” [MeSH] OR “prasugrel” [MeSH] OR “clopidogrel” [MeSH]. Furthermore, we searched the reference list of relevant guidelines, reviews, editorials, and studies on this topic. The literature screening process is summarized in Figure 1A.

Studies were considered eligible if they fulfilled all the following criteria: (1) Clinical studies with a prospective design, including patients who received DAPT schemes for the treatment of percutaneous coronary intervention. (2) Randomized studies comparing the clinical outcomes of a group of patients with P2Y12 inhibitor-based dual antiplatelet therapy. (3) Studies that evaluate the benefit of P2Y12 inhibitor monotherapy or switching to clopidogrel at a predefined time point ( ≤3 months), assisted by genetic testing, platelet function testing, or without.

### Quality assessment and endpoints

Two investigators (O.A.A and D.T) independently evaluated the titles and abstracts of all citations, in line with the PICOS criteria; any discrepancies were resolved by a third investigator (A.K.).

Articles, that met predefined eligibility criteria, were chosen for full-text screening and were reviewed by the two investigators against the eligibility criteria outlined in the PICOS framework: Patients who underwent coronary stent implantation (P), whether an intervention with dual antiplatelet abatement strategy with P2Y12 inhibitor monotherapy or P2Y12 inhibitor de-escalation to clopidogrel (I), compared with P2Y12 inhibitor plus aspirin dual antiplatelet therapy (C) has a favorable effect on bleeding, or major adverse cardiovascular events (MACE) or mortality (O).

The primary efficacy outcome of our analysis was the occurrence of MACE, defined as the composite of cardiovascular mortality, MI, and stroke. Major bleeding and all-cause mortality were assessed as main safety endpoints. Secondary outcomes included the individual components of MACE and stent thrombosis, defined according to the ARC criteria. Furthermore, safety outcomes, such as the frequency of major and minor bleeding complications, were also evaluated. In the case of the availability of multiple bleeding definitions, we extracted data according to the Bleeding Academic Research Consortium (BARC) criteria, defining type 3 or type 5 as major and type 2 as minor bleeding. The data were extracted, and the endpoints of interest were collected up to the 1st year after the coronary intervention.

The methodological qualities of the studies were also assessed using the Cochrane Collaboration tool for assessing the quality of RCTs.

### Data analysis

We pre-specified the use of multiple treatment network meta-analysis (NMA). The rates of events with each antiplatelet treatment combination were entered as an individual study arm, and data were pooled in a multiple treatment NMA that allows integration of direct and indirect comparisons. We calculated the risk ratio (RR) and its standard error using a frequentist approach to construct an NMA model accounting for the correlated treatment effects (8, 9). A random-effects model was applied by adding the estimated heterogeneity to the variance of each comparison, using an adaptation of the DerSimonian–Laird estimator. The random-effects model was chosen based on the consideration that the true preventive effect of antithrombotic treatment may vary from study to study and is influenced by the heterogeneity of the included trials. Values of *I*^2^ representing the amount of inconsistency, and Cochran's *Q* statistic and its corresponding *p*-value measuring the heterogeneity in the network were also calculated (8, 10).

Effect sizes are depicted as forest plots with potent dual antiplatelet therapy set as a reference. Furthermore, a comparative ranking of the treatments according to the *P*-scores method [a frequentist analog of SUCRA (Surface Under the Cumulative Ranking curve) was also performed (9)].

We appraised potential bias in the individual studies using the Cochrane Collaborations' bias assessment tool. To assess publication bias, a comparison-adjusted funnel plot supplemented with Eggers' test results was used (11).

The assumption of consistency; that the direct evidence for the effect size between two treatments in a network does not differ from the indirect evidence, was assessed by comparing and visualizing direct and indirect evidence.

Additional exploratory analyses included stratification and subgrouping based on the different de-escalation strategies and the included patient population, study size, and follow-up time.

Calculations were performed using R statistical software package version 4.0.3 (12), using the packages “meta 4.11-0,” “netmeta 1.2-0,” and “gemtc 0.8-4” (13). A *p*-value of < 0.05 was considered to represent statistical significance.

## Results

Ten studies that included 42,511 patients met the inclusion criteria. Among the included patients, 6,359 were randomized to a P2Y12 inhibitor de-escalation strategy, while 13,062 received potent P2Y12 inhibitor monotherapy. The included trials randomized patients treated with coronary intervention and stent implantation after an acute coronary syndrome event except for two studies where patients after a planned coronary intervention were also included. Potent P2Y12 inhibitor-based dual antiplatelet therapy control involved 18,540 cases while clopidogrel and aspirin combination involved 946. The characteristics and design of the included RCTs are shown in Table 1. The P2Y12 inhibitor de-escalation strategy was guided based on platelet function testing in two studies, based on genetic testing in two, and unguided, uniform in four. The size of the trials ranged from 131 to 15,968 participants, and the follow-up time was from 1 week to 12 months. The Global Leaders trial followed patients for 24 months after coronary intervention; however, as the patient received ticagrelor monotherapy or conventional DAPT during the 1st year, while during the 2nd-year, patients in the control received aspirin and in the experimental arm ticagrelor monotherapy, we extracted data from the first 12 months landmark analysis.

**Table 1:** Main characteristics of the included studies.

**First author**	**Claassens**	**Cuisset**	**Kim**	**Sibbing**	**Pereira**	**Ueno**	**Park**	**Kim**	**Mehran**	**Vranckx**
Publication year	2019	2017	2020	2017	2020	2016	2021	2020	2019	2018
Acronym	POPular Genetics	TOPIC	HOST-REDUCE-POLYTECH-ACS	TROPICAL-ACS	TAILOR-PCI	-	TALOS-AMI	TICO	TWILIGHT	GLOBAL LEADERS
Design	R open label	R, open label, single center	R, open label, multi-center	R, open label, multi-center	R, open label, multi-center	R, open label, multi-center	R, open label, multi-center	R, multi-center	R, open label	R, OPEN LABEL
Number of patients	2,751	646	2,338	2,610	5,302	131	2,590	3,056	7,119	15,968
Time between PCI and randomization	48 h	1 month	1 month	2 weeks	72 h	At the PCI	1 month	3 months	3 months	1 month
STEMI (%)	100	40	14	55	22	48	54	36	0	13
NSTEACS (%)	0	60	85.2	44	59	52	46	64	30	34
UAP (%)	0	NA	60	0	30	39	0	31.	70	13
CCS (%)	0	0	0	0	18	47.	0	0	35	47
Clopidogrel (experimental/control; %)	60.6/7.0	100/0	-	100/0	15/99	100/0	100/0	36/33	-	53/53.2
Prasugrel (experimental/control; %)	1 / 2.3	56/59	100/100	0/100	-	0/100	-	-	-	-
Ticagrelor (experimental/control; %)	38.1/90.5	44/42	-	-	85/1	-	0/100	73/70	0/100	47/46.8
Study group type	P2Y12-De	P2Y12-De	P2Y12-De	P2Y12-De	P2Y12-De	P2Y12-De	P2Y12-De	P2Y12-Mo	P2Y12-Mo	P2Y12-Mo
Definition of bleeding (primary/secondary)	PLATO/BARC	TIMI/BARC	BARC	BARC	BARC/TIMI	BARC/TIMI	BARC	TIMI	BARC/TIMI, GUSTO, and ISTH	BARC
End point	Bleeding, MACE, ST, and TVR	Bleeding, UREV, and MACE	Bleeding, TVR, MACE, and ST	Bleeding, MACE, UREV, and ST	CVD, MI, ST, stroke, and SRI	PRU	CVD, MI, stroke, and bleeding	Major bleeding, death, MI, ST, TVR, and stroke	Bleeding, MI, stroke, and death	Q-wave MI, and death
Follow-up, months	12	12	12	12	12	15	12	12	12	24
Age (mean ± SD)	61.7 ± 11.3	60.0 ± 10.2	58.8 (9.0)	58.7 (10.2)	62 (21–95)	68.8 ± 10.3	60 ± 11	61 (11)	65.01 ± 10.3	64.5 ± 10.3
Female, *N* (%)	317 (25.5)	114 (18)	251 (10.75)	2,052 (78.5)	1,738 (32.78)	32 (24.4)	454 (16.8)	628 (20.5)	1,698 (23.8)	3,714 (23.2)
DM, *N* (%)	288 (11.6)	177 (27)	990 (42.3)	527 (20)	1,938 (36.55)	53 (40.5)	731 (27.2)	835 (27)	2,620 (36.8)	4,038 (25.3)
Smoking, *N* (%)	1,127 (45.8)	286 (44)	838 (71.7)	1,182 (45)	1,752 (33.04)	NR	-	1,142 (37)	1,548 (21.7)	4,169 (26.2)
HTN, *N* (%)	1,032 (41.4)	313 (48)	1,476 (63.1)	1,599 (61.5)	4,409 (83.15)	89 (67.9)	1,318 (48.9)	1,541 (50.5)	5,154 (72.4)	11,705 (73.6)
DES, *N* (%)	NR	585 (91)	2,338 (100)	2,005 (77)	NR	NR	-	NR	NR	19,415 (94.6)
PCI approach (%)	NR	Femoral (4) Radial (96)	NR	NR	NR	NR	Femoral (49.4) Radial (49.4)	NR	NR	Femoral (26) Radial (74)

R, randomized; ACS, acute coronary syndrome; BARC, Bleeding Academic Research Consortium Criteria; DES, drug-eluting stent; DM, diabetes mellitus; HTN, hypertension; LD, loading dose; MD, maintenance dose; MACE, major adverse cardiac events; NR, not reported; O, observational study; R, retrospective; SD, standard deviation; ST, stent thrombosis; TIMI, Thrombolysis in Myocardial Infarction; TVR, target vessel revascularization; UREV, urgent revascularization; PLATO, Platelet Inhibition and Patient Outcomes; MI, Myocardial infarction; SRI, Severe Recurrent Ischemia; PRU P2Y12, Reaction Unit; STEMI ST, segment elevation MI; NSTEACS, non-ST-segment elevation acute coronary syndrome; UAP, unstable angina pectoris; CCS, chronic coronary syndrome; De, de-escalation; Mo, monotherapy.

Three trials used selective P2Y12 inhibitor de-escalation strategies. Among these, the POPular Genetics trial (5) and the TAILOR-PCI trial (14) used genetic testing with TaqMan assays. In the POPular Genetics trial, carriers of the loss-of-function CYP2C19 allele were treated with ticagrelor or prasugrel (49%), whereas non-carriers (CYP2C19^*^1/^*^1) received clopidogrel (51%). In the TAILOR-PCI trial, patients identified as possessing CYP2C19^*^2 or ^*^3 LOF alleles (CYP2C19 LOF carriers) were prescribed ticagrelor for maintenance therapy or prasugrel for patients who did not tolerate ticagrelor, and non-carriers or those with inconclusive results were prescribed clopidogrel.

In the TROPICAL-ACS trial (6), a platelet-function testing-based de-escalation treatment algorithm was applied. Patients in the P2Y12 inhibitor de-escalation group received a post-discharge treatment consisting of 1-week prasugrel treatment (10 or 5 mg per day) followed by 1 week of clopidogrel treatment (75 mg per day) and a platelet function measurement (on clopidogrel) 2 weeks after hospital discharge (PFT-guided de-escalation group). The network of evidence, both regardless of, and with regard to the applied de-escalation strategies, is depicted in Figures 1B, C.

The risk of bias was assessed for all the trials, showing a minimal risk in all biases. The results derived from direct comparisons were identical to those computed with the help of indirect comparisons (Supplementary Figures 1–3).

**Figure 2:**
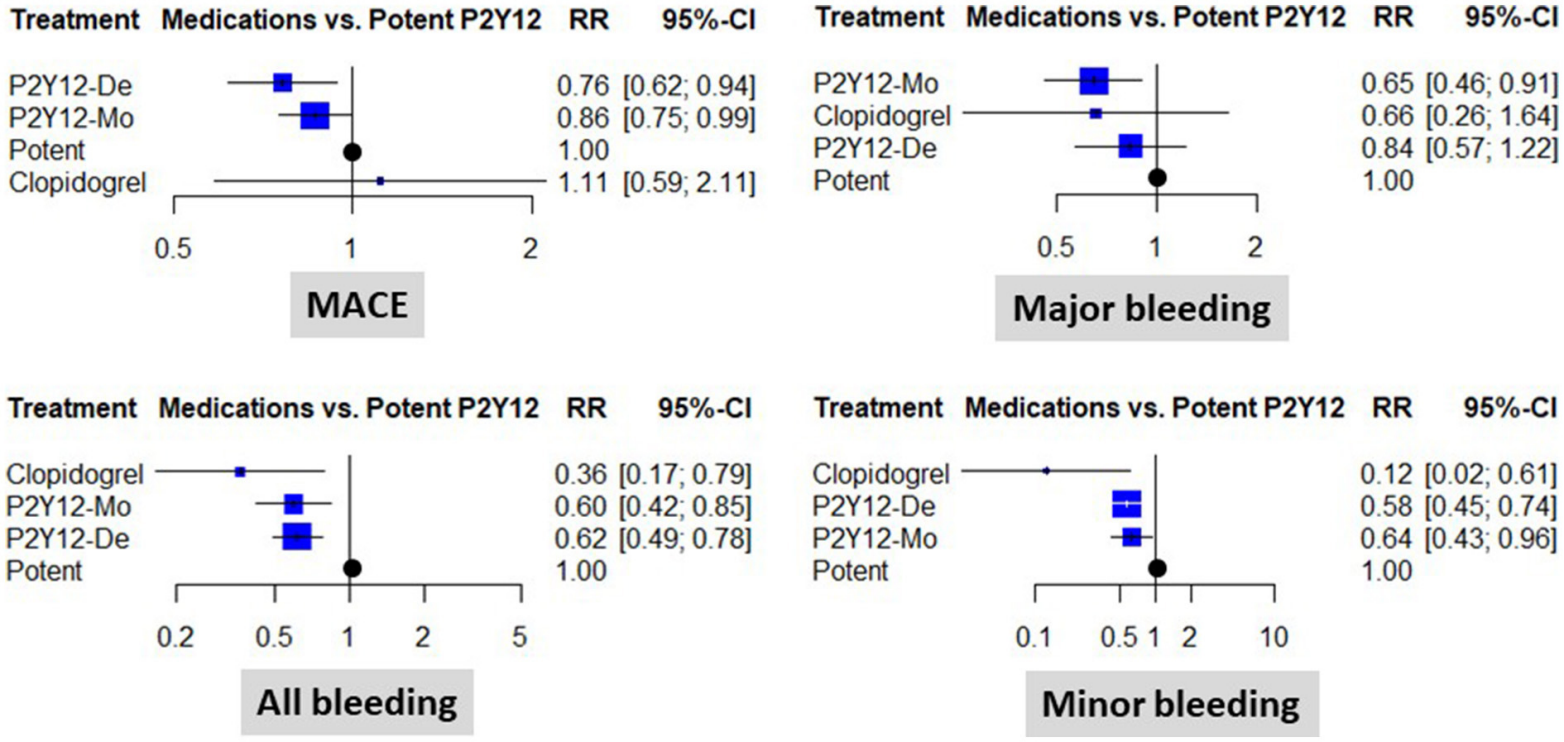
Clinical results of using different abatement strategies. The forest plots depict the results of the network meta-analysis computed based on direct and indirect comparisons as risk ratio (RR) and 95% confidence intervals (95% CI). Data are presented as compared to the potent P2Y12 inhibitor-based dual antiplatelet therapy (marked as “Potent). MACE, major adverse cardiovascular events; P2Y12-De, P2Y12 inhibitor de-escalation; P2Y12-Mo, potent P2Y12 inhibitor monotherapy; Clopidogrel, clopidogrel based DAPT.

When compared to a potent dual antiplatelet strategy, both P2Y12 inhibitor de-escalation and P2Y12 inhibitor monotherapy were associated with a significant ischemic risk reduction. The estimated cumulative effect reached a 24% risk reduction with P2Y12 inhibitor de-escalation and a 14% risk reduction with P2Y12 inhibitor monotherapy [RR: 0.76 (0.62, 0.94), *p* < 0.05, and RR: 0.86 (0.75, 0.99), *p* < 0.05, respectively]. The results were consistent without important heterogeneity (*p* = 0.91 within designs), and the *I*^2^ test showed low levels of inconsistency (between designs): *I*^2^ = 0% (0.0%; 17.6%) (Figure 2).

**Figure 3:**
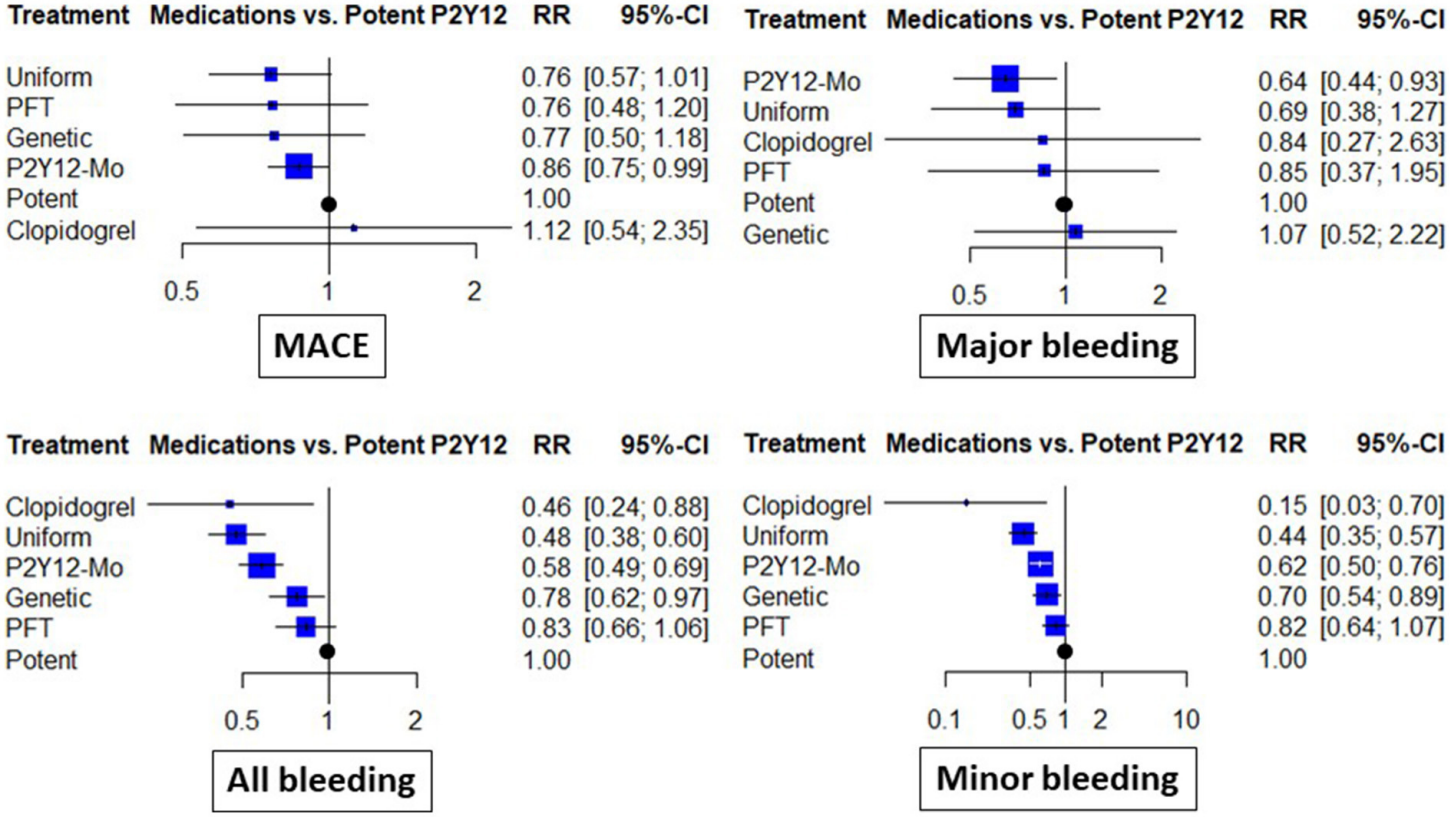
Clinical results of abatement strategies considering de-escalation strategies separately. The forest plots depict the risk ratio (RR) and 95% confidence interval (95% CI) achieved with the abatement strategies compared to the potent P2Y12 inhibitor-based dual antiplatelet therapy for major adverse cardiovascular events (MACE), all bleeding (including major and minor events), as well as major bleeding and minor bleeding. In these analyses, de-escalation strategies were considered separate subgroups based on the use of genetic or platelet-function (PFT) testing guidance or uniform de-escalation. P2Y12-Mo, potent P2Y12 inhibitor monotherapy; Clopidogrel, clopidogrel based DAPT.

When different de-escalation strategies were considered, a similar tendency for risk reduction was observed; however, this association did not reach the level of statistical significance in any case (Figure 3).

Individual components of the composite endpoint showed beneficial trends, with a lower risk of ischemic events in the abatement strategies except for the risk of myocardial infarction, stent thrombosis, and stroke. These showed an increased risk after P2Y12 inhibitor monotherapy; however, none of these differences reached the level of statistical significance (Supplementary Figure 4).

Treatment ranking gave the highest rank to P2Y12 inhibitor de-escalation (0.92), followed by P2Y12 inhibitor monotherapy (0.62), and the lowest to the clopidogrel or potent P2Y12 inhibitor-based dual antiplatelet therapy (0.24 and 0.22, respectively) in terms of MACE. P2Y12 inhibitor monotherapy (0.78) ranked higher than clopidogrel (0.67) and P2Y12 inhibitor de-escalation (0.42) as well as potent P2Y12 inhibitor-based-dual antiplatelet therapy (0.12) in terms of major bleeding.

Major bleeding rates were similar between P2Y12 inhibitor de-escalation and the control, without major differences among trials [RR: 0.84 (0.57, 1.22)]; however, P2Y12 inhibitor monotherapy resulted in a 35% reduction [RR: 0.65 (0.46, 0.91), *p* < 0.05, *I*^2^ = 0%]. Differences were more expressed in the analyses of all bleeding events and were substantially influenced by minor bleeding. Both P2Y12 inhibitor de-escalation and P2Y12 inhibitor monotherapy resulted in a 36–42% reduction (Figure 2). The most expressed reduction was observed for uniform de-escalation, followed by the other strategies. In the case of PFT-guided de-escalation, no bleeding endpoint was significantly reduced (Figure 3).

Each comparison between de-escalation and monotherapy resulted in an effect estimate that did not reach the level of statistical significance. When considering, however, the different subgroups of de-escalation strategy results, with uniform de-escalation, the estimates were similar to that of monotherapy, while the rates of minor and major bleeding were significantly higher than that for monotherapy (Supplementary Table 1).

Leave-one-out sensitivity exercises did not show any signal of individual studies having excessive influence in the network (Supplementary Figure 5). Further subgroup analyses supported the consistency of the findings (Supplementary Figure 6).

## Discussion

In this network meta-analysis of DAPT abatement strategies, we found that both switching to a less potent P2Y12 inhibitor, with a P2Y12 inhibitor de-escalation strategy, or using potent P2Y12 monotherapy with aspirin cessation, were associated with better results with regard to the ischemic endpoints. Benefits in terms of bleeding risk reduction were also associated with both strategies; however, reduction of major bleeding was only significant with P2Y12 monotherapy.

Bleeding events represent an important Achilles' heel of adjunctive pharmacotherapy after coronary interventions. To improve prognosis, bleeding avoidance strategies are widely applied and include both pharmacological and non-pharmacological approaches. The benefits of intensified antiplatelet therapy were demonstrated in cases with the highest ischemic risks as well as in the timeframe closest to the intervention. However, as time passes, this advantage may be overweighted by the cumulative risk of bleeding. Multiple trials were conducted to test alternative protocols, with the potential to attenuate long-term bleeding risk. In a comprehensive analysis of these recent studies, we found that abatement from a potent P2Y12 inhibitor-based dual antiplatelet treatment was associated with an important reduction of bleeding events in patients treated with PCI. Both strategies, with de-escalation of P2Y12 inhibitor and P2Y12 inhibitor monotherapy, showed advantages; however, the analysis also explored important differences which have potential practical implications. While both strategies reduced the risk of all bleeding, P2Y12 inhibitor monotherapy, but not P2Y12 inhibitor de-escalation schemes, was associated with a significant reduction of major bleeding events. Our analysis also suggests that this benefit is not counterbalanced with a higher risk of ischemic events. Nonetheless, the individual trials showed only beneficial trends; this was associated with a significant reduction only in the cumulative analyses. These findings suggest routine use of abatement in patients with ACS undergoing PCI in the early phase. If applied according to the trials, i.e., between 48 h and 3 months, these strategies could be beneficial in terms of improvement of ischemic and bleeding risk.

The three oral P2Y12 inhibitors currently used in patients with ACS and PCI exhibit important pharmacodynamic and pharmacokinetic differences. Clopidogrel and prasugrel are prodrugs that are transformed into their active metabolites by hepatic cytochrome P450 enzymes (15). This activation step is faster and more effective in the case of prasugrel, and the active metabolite of both substances irreversibly inhibits the P2Y12 receptor on platelets. Ticagrelor reversibly inhibits the binding of ADP to the P2Y12 receptor in a non-competitive manner. Ticagrelor is an active drug that does not require *in vivo* biotransformation (16). Compared with clopidogrel, both alternatives have faster onsets, are more potent, and have less response variabilities (17).

One of the main limitations of clopidogrel is that the achieved platelet function inhibition reflects high-interindividual variability, which, among high-risk patients, also represents an important risk marker (18). High-platelet reactivity can be verified with the help of platelet function testing and is present in a higher frequency among mutation carriers of cytochrome enzymes involved in thienopyridine metabolism. These include CYP2C19 mutant alleles such as loss-of-function CYP2C19^*^2 and ^*^3 alleles. Carriers of these two non-functional copies of the CYP2C19 gene are classified as CYP2C19 poor metabolizers and are characterized by a reduced efficacy of clopidogrel. Other variations include the CYP2C19^*^17 gain-of-function allele, which can be found in rapid clopidogrel metabolizers. Due to genetics and the high rate of potential drug interactions, there is large interindividual variability in response to clopidogrel, and 15–40% of individuals, depending on the criteria used, are considered “non-responders,” or “clopidogrel-resistant,” with high residual platelet aggregation. There is a vast amount of evidence indicating that high-platelet reactivity, despite clopidogrel treatment, is a risk factor for cardiovascular events and stent thrombosis, while lower levels of residual platelet aggregation are associated with a higher frequency of bleeding complications (19).

While P2Y12 inhibitor monotherapy was associated with a significant reduction of both major bleeding and adverse events, the effects of P2Y12 inhibitor de-escalation strategies were different. The cumulative ischemic risk reduction was more expressed with these strategies; however, despite favorable tendencies, only the risk of minor bleeding was significantly reduced. All three P2Y12 inhibitor de-escalation strategies resulted in a similarly lower rate of ischemic events; the reduction of bleeding events was most associated with uniform de-escalation. Guided de-escalation with platelet function genetic testing showed less expressed reduction of the bleeding endpoints.

Therefore, P2Y12 inhibitor de-escalation strategies seem to be more efficient in decreasing ischemic risk, while P2Y12 inhibitor monotherapy is a safer strategy for reducing bleeding in patients with ACS. However, using ticagrelor in the P2Y12 inhibitor monotherapy strategy could lead to lower ischemic risks than clopidogrel (20).

While abatement strategies reduced the rate of MACE and bleeding compared to potent P2Y12-based DAPT, indirect comparisons of P2Y12 inhibitor monotherapy and de-escalation only explored signals that may guide decision-making. The reduction of bleeding was similar between the two alternatives; however, subgroup analyses showed that genetic testing and platelet function test-guided de-escalation strategies lagged behind P2Y12 inhibitor monotherapy. This suggests that if bleeding reduction is the main interest, P2Y12 inhibitor monotherapy or unguided de-escalation may offer better alternatives. In indirect comparisons of the rate of ischemic events, however, a tendency for an 11–12% reduction with P2Y12 inhibitor de-escalation strategies was observed; these differences did not reach the level of statistical significance. Thus, more data is required to inform ischemic risk reduction-based decision-making.

Both pivotal clinical trials verifying the benefits of prasugrel and ticagrelor over clopidogrel in ACS showed a reduction of recurrent ischemic events with more effective P2Y12 inhibition but counterbalanced with some degree increase of bleeding risk. The importance of bleeding reduction strategies in ACS was recently emphasized (20, 21). Moreover, because of the publication of alternative antiplatelet protocols, multiple meta-analyses were published. Our meta-analysis differs from these in several aspects (22). Guo et al. (23) included in their meta-analysis both randomized and observational studies. In addition to updating the literature search to include the latest trials, we restricted our inclusion criteria to randomized controlled studies. As observational trials suffer from multiple downsides due to inclusion bias, we considered excluding them to improve the robustness of our analysis. Angiolillo et al. (24) included in their meta-analysis only studies of de-escalation from ticagrelor to clopidogrel, while our meta-analysis also includes de-escalation from both potent P2Y12 inhibitors to clopidogrel. A number of studies focused on the outcomes and benefits of guided de-escalation. Galli et al. (25) found that guided de-escalation improved both composite and individual efficacy outcomes and that it is associated with the most favorable balance between safety and efficacy (26). Tavenier et al. (27) presented results that suggest that both guided and unguided de-escalation were associated with lower rates of bleeding and ischemic events, which aligns with our results. However, the latter meta-analysis excluded aspirin monotherapy trials, which were included in this meta-analysis. Furthermore, with the inclusion of trials testing P2Y12 inhibitor monotherapy and P2Y12 inhibitor de-escalation, our analysis enables the comparison of different abatement strategies.

Thus, far, many randomized controlled trials have investigated the optimal duration of DAPT and meta-analyses comparing different DAPT lengths (3, 6, 12, 24, or 30 months) following DES implantation. The association of prolonged DAPT with an increased bleeding risk, along with a potential reduction of recurrent myocardial infarction (MI) and ST, has been assessed. In an NMA of these trials, D'Ascenzo et al. found that the type of stent impacts the risk of adverse events in addition to DAPT duration. However, there is limited data that directly compare different DAPT durations in patients treated with different generation DES or bioresorbable scaffolds.

Earlier analyses in line with our results reported that P2Y12 inhibitor de-escalation reduces ischemic risk and bleeding in patients with ACS. We extended these observations, with a similar reduction observed in the P2Y12 inhibitor monotherapy trial. Our analysis also enabled comparison of the two strategies. Our results align with the outcomes of the recent meta-analyses by Laudani et al. (28) and Ullah et al. (29), where P2Y12 inhibitor de-escalation decreased ischemic risk, and P2Y12 inhibitor monotherapy decreased bleeding.

## Limitations

This meta-analysis has some limitations such as differences in the definition and adjudication of clinical outcomes, diverse follow-up duration, and inconsistency in the timing of switching. Also, few trials were identified, and the low number of events was a typical characteristic of the included studies. Not all studies restricted their inclusion to patients with ACS; however, when relative risk measures are used, differences in absolute risk are less influential to a network. Thus, neither exclusion nor subgroup analyses reflected an important influence attributable to the inclusion of a lower-risk population. We still support the need for adequately powered RCTs to evaluate de-escalation and to further elucidate the role of risk stratification, including potential genetic and PFT characteristics, before applying antiplatelet abatement. It is important to underline that several treatment combinations were not directly compared in specifically designed trials, and thus, an important part of the effect estimates are only based on indirect comparisons. Furthermore, the inclusion of multiple treatment options may also weaken the consistency of the analysis. Thus, the results should be interpreted as observational and only hypothesis-generating.

A new randomized study, the ELECTRA-SIRIO 2 study, which is still underway, aims to evaluate the safety and efficacy of two ticagrelor-based de-escalation antiplatelet strategies in patients with ACS. The results of this study could help inform and confirm the benefits of de-escalation.

Despite these limitations, this systematic review, with a meta-analysis, provides robust evidence evaluating the risks and benefits of abatement strategies.

## Conclusion

Our findings suggest that the abatement of antiplatelet treatment gives better results in terms of the bleeding risk, without compromising the major adverse cardiovascular events risk, which turns out to be significantly lower. P2Y12 inhibitor monotherapy and P2Y12 inhibitor de-escalation exhibit differences that may influence their clinical use. P2Y12 inhibitor monotherapy resulted in a reduction of both major and minor bleeding, while ischemic risk reduction was less expressed. The de-escalation strategy was quite the opposite, as there was no difference in major bleeding between this strategy and the control; however, ischemic risk was strongly reduced. Despite their plausible background data, trials with guided de-escalation showed less expressed benefits. It is of note that, in selected patients with high-ischemic risk, these strategies may still offer a safe alternative compared to the long-term potent P2Y12 inhibitor DAPT.

## Impact on daily practice

Dual antiplatelet therapy, using a potent P2Y12 inhibitor in patients with acute coronary syndrome receiving percutaneous coronary intervention, maintained for up to 12 months is a guideline-recommended therapy.

Alternative abatement schemes may improve safety outcomes such as major bleeding, without increasing the frequency of ischemic endpoints, creating an optimal balance between bleeding and ischemic complications.

P2Y12 inhibitor monotherapy significantly reduced both major and minor bleeding, while with P2Y12 inhibitor de-escalation, only minor bleeding risk was reduced. Both strategies also significantly reduced the rate of ischemic complications.

## Data availability statement

The original contributions presented in the study are included in the article/Supplementary material, further inquiries can be directed to the corresponding author.

## Author contributions

OE and DT performed the literature search and the data extraction. DT and AK performed the statistical analysis. All authors participated in the conception and drafting the manuscript. All authors contributed to the article and approved the submitted version.
