# Supplementary material for: Abatement of potent P2Y12 antagonist-based dual antiplatelet therapy after coronary intervention: A network meta-analysis of randomized controlled trials
Source: Front Cardiovasc Med. 2023 Jan 12;9:1008914. doi: 10.3389/fcvm.2022.1008914 (PMC9877531; doi:10.3389/fcvm.2022.1008914)

# Supplementary material

##### to the article entitled “Abatement of potent P2Y12 based antagonist dual antiplatelet therapy after coronary intervention– a network meta-analysis of randomized trials”

Contents

[Supplementary material 1](#_Toc123666665)

[Figure S1 Assessment of publication bias. 2](#_Toc123666666)

[Figure S2 Assessment of bias. 3](#_Toc123666667)

[Figure S3 Visualizing direct and indirect evidence in the entire network (Panel A) and in subgroups according to de-escalation strategies (Panel B). 5](#_Toc123666668)

[Figure S4 Results of the network analysis of ischemic endpoints. 7](#_Toc123666669)

[Table S1 Results of P2Y12 inhibitor monotherapy compared to P2Y12 de-escalation strategies. 8](#_Toc123666670)

[Figure S5 Results of the leave-one-out sensitivity exercises. 9](#_Toc123666671)

[Figure S6 Results of the subgroup analyses. 10](#_Toc123666672)

## Figure S1 Assessment of publication bias.

Comparison-adjusted funnel plot showed no signs of important publication bias. *Abbreviations*: P2Y12-De; P2Y12 inhibitor de-escalation, P2Y12-Mo; potent P2Y12 inhibitor monotherapy


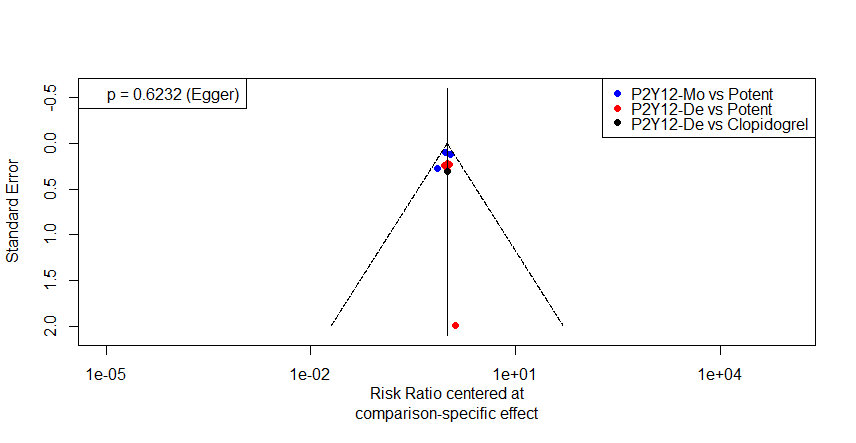


## Figure S2 Assessment of bias.

The chart shows the individual (Panel A) and the summarized results (Panel B) of the bias assessment of included trials using the Cochrane bias assessment tool. Of note is that in none of the studies was complete treatment blinding implemented, however, as the outcome was not directly influenced per the Cochrane Collaboration user instructions we evaluated detection bias as low risk of bias.

No blinding or incomplete blinding was evaluated

##### A
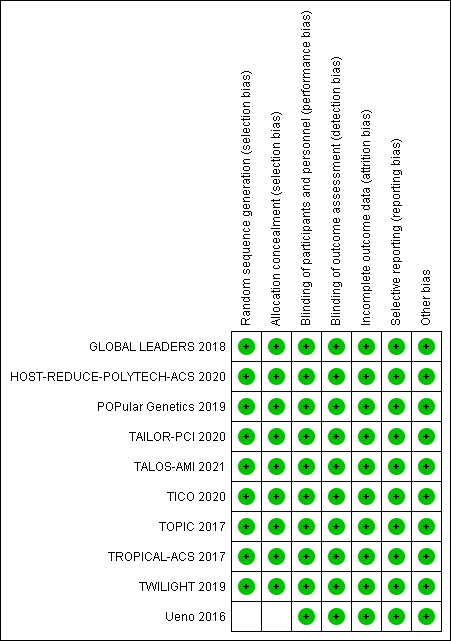


##### B
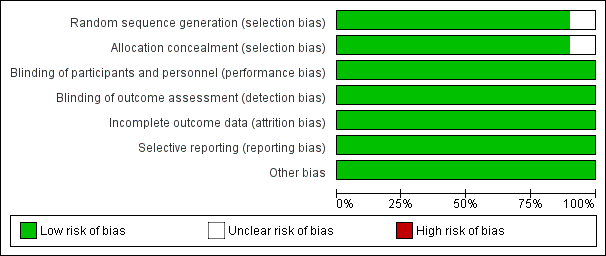


## Figure S3 Visualizing direct and indirect evidence in the entire network (Panel A) and in subgroups according to de-escalation strategies (Panel B).

##### The proportion of direct and indirect evidence used to estimate each comparison is depicted. The plot also provides two additional metrics: the minimal parallelism and mean path length of each estimated comparison. According to König, Krahn, and Binder (2013), a mean path length > 2 means that a comparison estimate should be interpreted with caution.

Panel A


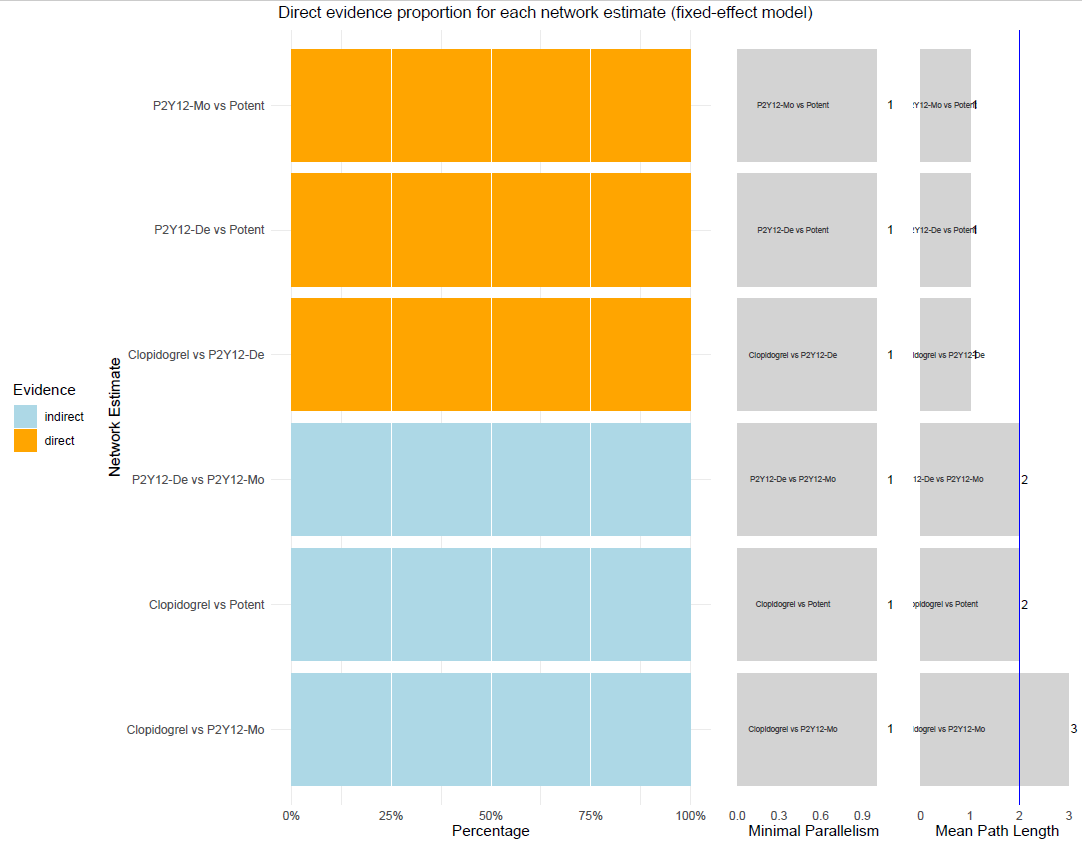


Panel B
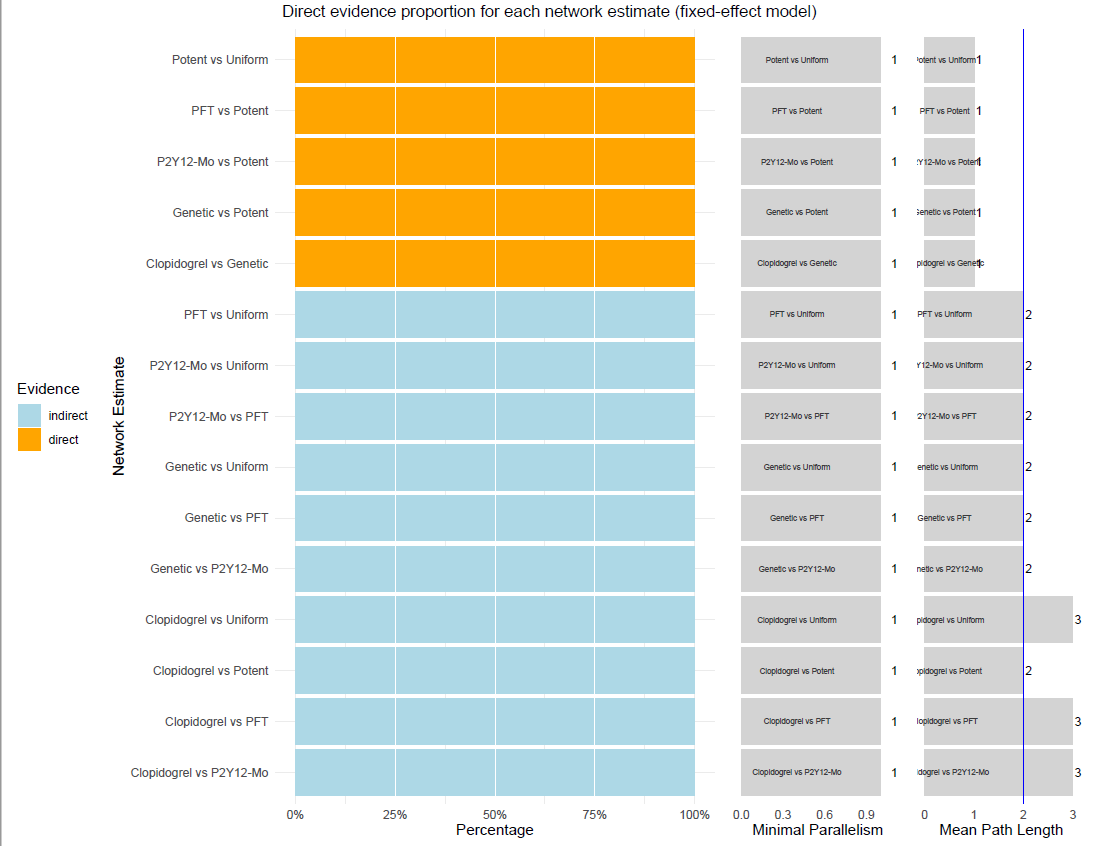


## Figure S4 Results of the network analysis of ischemic endpoints.

##### The forest plot depicts risk ratio and 95% confidence interval with the abatement strategies compared to the potent P2Y12 inhibitor based dual antiplatelet therapy. Abbreviations: MACE: major adverse cardiovascular events, P2Y12-De; P2Y12 inhibitor de-escalation, P2Y12-Mo; potent P2Y12 inhibitor monotherapy

#####
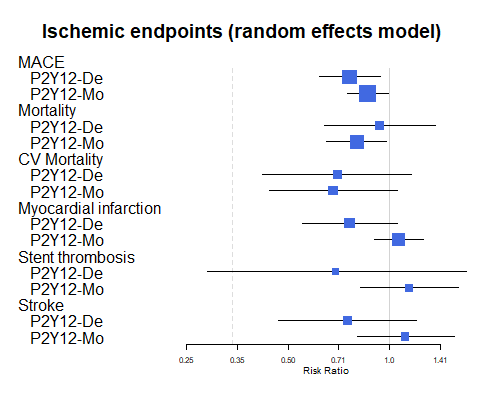


## Table S1 Results of P2Y12 inhibitor monotherapy compared to P2Y12 de-escalation strategies.

The table depicts risk ratio and 95% confidence intervals computed from the network indirect comparisons. Clinical endpoints in comparisons with de-escalation as a group, did not show significant differences (first Column). Among the different de-escalation strategies both genetic testing guided (Genetic) and platelet function guided (PFT) de-escalation lagged behind P2Y12 monotherapy in terms of all bleeding (minor and major). (Columns 2-4.) Abbreviations: MACE: major adverse cardiovascular events, CV Death: cardiovascular mortality, MI: myocardial infarction, PFT: platelet function testing.

|  | De-escalation | Genetic | PFT | Uniform |
| --- | --- | --- | --- | --- |
| MACE | 0.88 (0.68; 1.13) | 0.89 (0.57; 1.39) | 0.88 (0.55; 1.42) | 0.88 (0.64; 1.21) |
| All Bleeding | 1.03 (0.68; 1.57) | **1.33 (1.00; 1.75)** | **1.43 (1.06; 1.91)** | 0.82 (0.62; 1.09) |
|  |  |  |  |  |
| CV Death | 1.04 (0.53; 2.02) | 1.34 (0.49; 3.63) | 1.15 (0.39; 3.39) | 0.80 (0.33; 1.96) |
| MI | 0.71 (0.50; 1.03) | 0.69 (0.37; 1.27) | 0.81 (0.46; 1.42) | 0.64 (0.35; 1.17) |
| Stroke | 0.67 (0.38; 1.19) | 0.66 (0.25; 1.72) | 0.39 (0.10; 1.55) | 0.76 (0.39; 1.50) |
| Mortality | 1.17 (0.76; 1.80) | 1.26 (0.65; 2.45) | 1.15 (0.50; 2.68) | 1.10 (0.60; 2.04) |
| Stent Thrombosis | 0.61 (0.24; 1.56) | 0.59 (0.09; 3.61) | 0.58 (0.09; 3.60) | 0.63 (0.17; 2.27) |
|  |  |  |  |  |
| Major Bleeding | 1.29 (0.78; 2.14) | 1.67 (0.74; 3.78) | 1.33 (0.53; 3.29) | 1.08 (0.53; 2.20) |
| Minor Bleeding | 0.90 (0.56; 1.44) | 1.13 (0.82; 1.56) | 1.33 (0.96; 1.86) | 0.72 (0.52; 0.99) |

## Figure S5 Results of the leave-one-out sensitivity exercises.

The forest plots depict the results of the random effect network analyses of the risk of major adverse event (MACE) (Panel A) and major bleeding (Panel B). Data are presented as relative risk and 95% confidence interval (RR [95%CI]) compared to the potent P2Y12 inhibitor based dual antiplatelet therapy in the full model and in analyses performed with individual studies ignored.

A
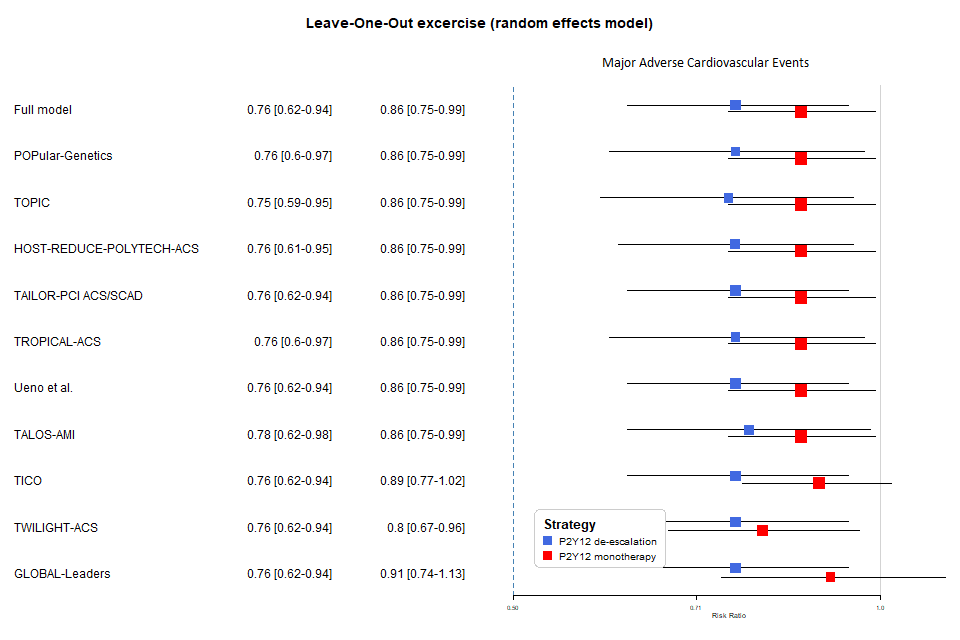


B
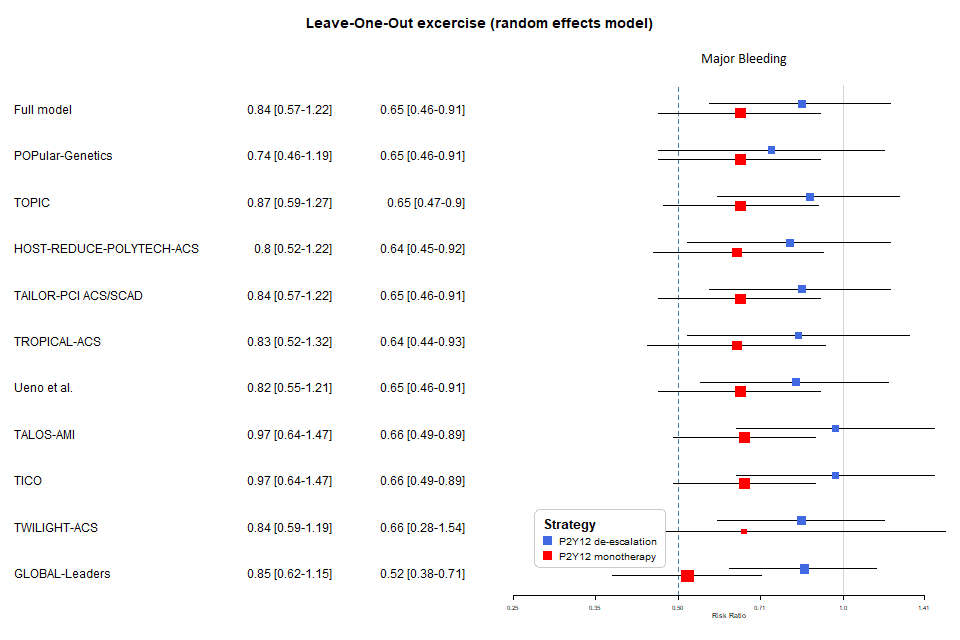


## Figure S6 Results of the subgroup analyses.

The forest plots depict the results of the random effect network analyses of the risk of major adverse event (MACE) (Panel A) and major bleeding (Panel B). Data are presented as relative risk and 95% confidence interval (RR [95%CI]) compared to the potent P2Y12 inhibitor based dual antiplatelet therapy. *Abbreviations*: CCS: chronic coronary syndrome, ACS: acute coronary syndrome, NA: not available, BARC: Academic Research Consortium bleeding definition

A
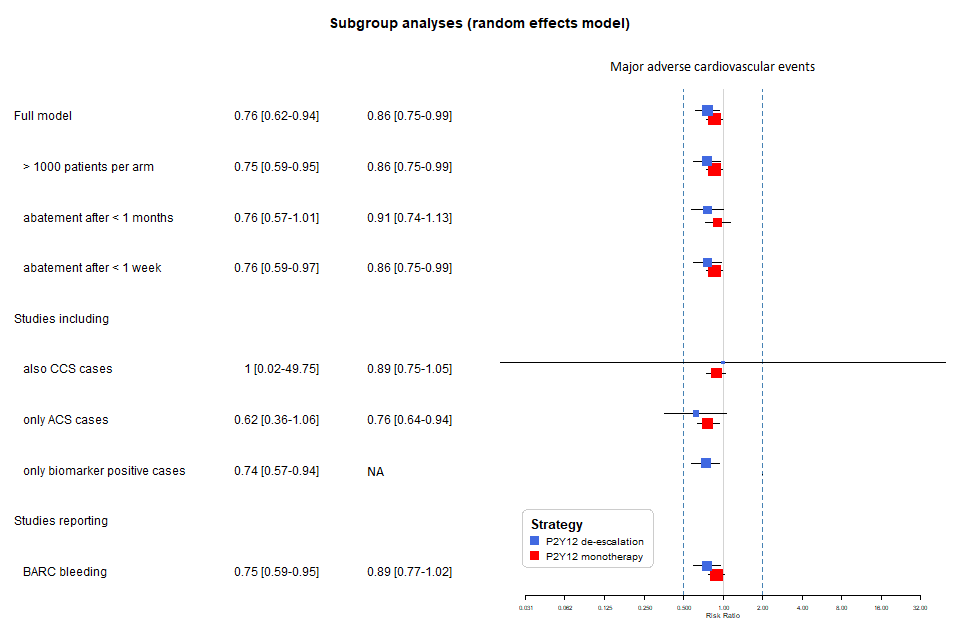


B
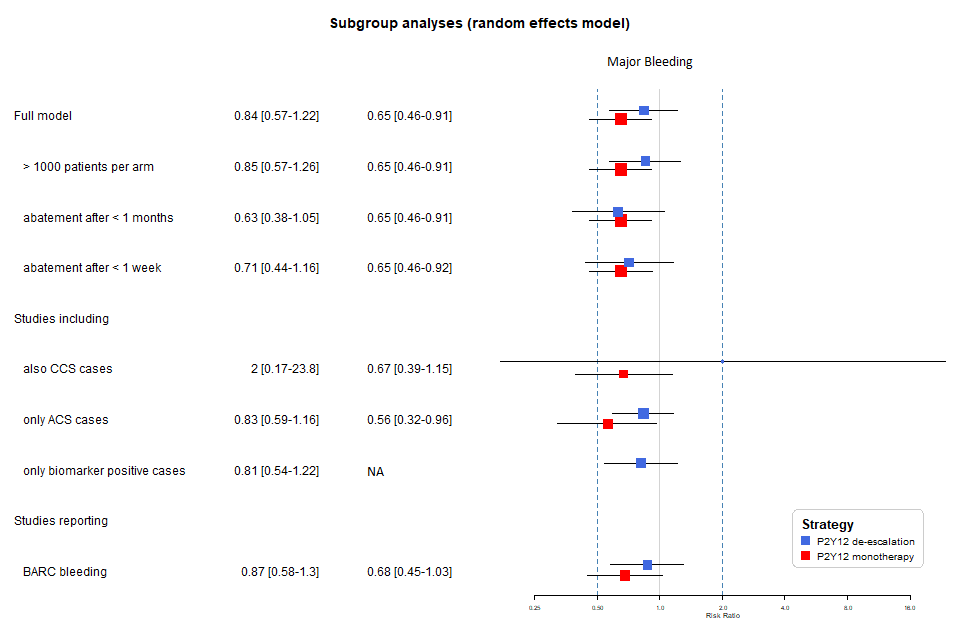

Supplement: Supplementary file 1 [file Data_Sheet_1.docx]
